# Differences in energy and nutrient content of menu items served by large chain restaurants in the USA and the UK in 2018

**DOI:** 10.1017/S1368980022001379

**Published:** 2022-06-01

**Authors:** Yuru Huang, Thomas Burgoine, Dolly RZ Theis, Jean Adams

**Affiliations:** MRC Epidemiology Unit, University of Cambridge School of Clinical Medicine, Box 285 Institute of Metabolic Science, Cambridge Biomedical Campus, Cambridge CB2 0QQ, UK

**Keywords:** Chain restaurants, Cross-country comparison, Energy, Nutrients, Restaurant food environment, Childhood obesity

## Abstract

**Objective:**

To quantify the sector-wide energy and nutritional differences of both adult and children’s restaurant menu items in the UK and the USA in 2018.

**Design:**

Cross-sectional study.

**Setting:**

Energy and nutritional information provided on restaurant websites.

**Participants:**

Menu items (*n* 40 902) served by forty-two large UK chains and ninety-six large USA chains.

**Results:**

Mean absolute energy, fat and saturated fat values were higher in USA menu items. For example, the mean adjusted per-item differences of adult menu items between the USA and the UK were 45·6 kcal for energy and 3·2 g for fat. Comparable figures for children’s menu items were 43·7 kcal and 4 g. Compared with UK menu items, USA adult menu items also had higher sugar content (3·2 g, 95 % CI (0·5, 6)), and children’s menu items had higher Na content (181·1 mg, 95 % CI (108·4, 253·7)). Overall, 96·8 % of UK and 95·8 % of USA menu items exceeded recommended levels for at least one of Na, fat, saturated fat or sugars.

**Conclusions:**

Menu items served by large chain restaurants had higher mean absolute levels of energy, fat and saturated fat in the USA compared with the UK. UK adult menu items were also lower in sugars compared with the USA ones and children’s items lower in Na. As more than 95 % of all items were considered to have high levels of at least one nutrient of public health concern in the USA and the UK, improvements in restaurant menu items are needed in both countries.

Eating food prepared out of the home has become increasingly common worldwide. The past decade has seen a sharp increase in ‘out-of-home’ food expenditure. Americans spent 45·2 % of total food expenditures on out-of-home food in 2020^(1)^. In the UK, 31·0 % of total food and drink expenditure was spent on eating out in 2018–2019^(2)^. Restaurant sales were projected to increase by 4 % in the USA in 2020 – despite the COVID pandemic – to a total of $899 billion^(3)^.

Out-of-home food is consistently more energy dense and lower in nutritional quality compared with food prepared at home^(4)^. Studies have indicated that more frequent consumption of restaurant food is associated with higher intakes of energy and nutrients detrimental to health, poorer overall diet quality and greater BMI^(5–7)^. Restaurant meals in both the UK and USA contain high levels of energy and macronutrients, such as total fat, carbohydrates and Na^(8–10)^. Only 8 % of meals offered at large USA chain restaurants met all seven healthy criteria set by the American Heart Association, and only 9 % of meals offered at large UK chain restaurants met guidelines for energy set by Public Health England^(8,11)^.

Consumption of restaurant foods is also an important contributor to childhood obesity^(12,13)^. Previous research indicates that children’s menu items from chain restaurants are high in energy, fat, saturated fat and Na^(14,15)^. Approximately one-third of children’s main dishes at fast-food restaurant chains in the USA, and the majority of those at sit down restaurant chains exceeded the 2010 Dietary Guidelines for children in 2010–2014^(15)^, with little evidence of improvement over time^(10)^. Likewise, a study in the UK found that 68 % of younger children’s (aged 2–5 years) and 55 % of older children’s (aged 6–12 years) meals contained more total fat than recommended^(14)^.

One potential explanation for the lack of improvement in the nutritional content of both adult and children’s menu items may be challenges of food technology. For example, ingredients that contain Na act as preservatives, leavening agents or emulsifiers and also help maintain the taste of foods^(16)^. Reducing Na content, therefore, requires Na alternatives to address food safety concerns while sustaining taste. Reducing other nutrients in food poses similar food science challenges^(17,18)^. However, there is substantial variability of menu item nutritional profiles in different countries, even for the same menu item at the same chain restaurant, indicating that there may be room for improvement despite food technology challenges^(19)^.

Yet while there is extensive research on the restaurant food environment in the USA, there is limited evidence on how it compares to other countries^(10,11,20)^. This includes the UK, which is generally thought to be culturally similar and has many chain restaurants in common. However, the UK and the USA differ in both obesity prevalence (42 % in the USA and 28 % in the UK in 2018), frequency of use of OOH food sources and policy context^(21–24)^. Previous studies of restaurant foods in both countries have highlighted individual product-level differences. As an example, the energy density of a Big Mac was 958 kcal/100 g in the UK and 1054 kcal/100 g in the USA in 2012^(19)^. These studies, however, focused on fast-food restaurant chains only and included relatively small samples of items. To date, no study has quantified differences in energy and nutrient content of whole menus, including those for adults and children, across all types of large chain restaurants and across countries.

In this study, we aimed to capture, quantify and compare the nutritional landscape of restaurant foods in the UK and USA, by comparing nutritional profiles of chain restaurant menu items in these two countries. We examined the energy and nutritional differences of both adult and children’s menu items separately.

## Methodology

This is a cross-sectional study. We collated energy and nutrient information published online for menu items served by large chain restaurants in the UK and the USA, in 2018. We used the data to examine energy and nutritional differences in menu items in these two countries and estimated the proportion of menu items exceeding recommended levels of Na, total fat, saturated fat and free sugars.

### Data collection

We acquired the 2018 USA data from the MenuStat project, a publically available nutritional database of food served by the largest chain restaurants in the USA^(25)^. We collected 2018 UK data as part of the UK MenuTracker project. Data collection methodologies of MenuStat (USA) and MenuTracker (UK) are comparable, and details of data collection are reported elsewhere^(26,27)^. Briefly, the largest 100 chains (based on total sales volume) in each country that posted online nutritional information in 2018 were included. Item-level nutritional information was manually transcribed from chain websites into Excel spreadsheets.

### Data standardisation

We standardised pizza portions because restaurants presented the information for this type of food differently. For example, some restaurants presented energy and nutritional information based on one slice of pizza (not always clarifying how many slices were in a pizza), while others presented information based on the whole pizza. For pizzas described in menus as medium, large, family sized or for sharing, we calculated the energy and nutrient content of three slices of pizza. For pizzas described as small or for individual consumption, the energy and nutrient content were calculated based on the whole pizza. This was in accordance with how Domino’s, a leading pizza chain, presented the energy and nutritional information on their pizzas. Salt (g) content was converted to Na (mg) with a conversion factor of 400. All column names were standardised before combining data from the two databases.

### Item- and restaurant-level characteristics

Restaurant chains were categorised as ‘fast-casual’, ‘fast-food restaurant’ or ‘full-service’, based on criteria defined previously^(20)^. Briefly, full-service restaurants were those that provide table service (e.g. Applebee’s in the USA, Zizzi in the UK). Fast-casual restaurants were those that self-identified as fast-casual, or met at least two of the following criteria, as defined previously^(28)^: no table service, food preparation onsite, commitment to higher quality/fresher ingredients or sustainability and reusable utensils (e.g. Starbucks). The commitment to higher quality/fresh ingredients or sustainability was identified through the company’s official website. Fast-food restaurants were those that provided no table services and met fewer than two of the criteria above (e.g. McDonald’s). In both data sets, MenuTracker and MenuStat, the following binary encoded features were available for every menu item: children’s menu items, limited time offer, regional and shareable status. Descriptions and examples for these features are described elsewhere^(20,26,27)^. In short, menu items were categorised based on descriptions provided by restaurant websites. Children’s menu items were items on ‘children’s’ or ‘kid’s’ menus or labelled in other ways as for ‘children’ or ‘kids’, and adult menu items were those not identified as children’s menu items. Each menu item was also coded into one of the twelve food categories: Appetisers & Sides, Baked Goods, Beverages, Burgers, Desserts, Fried Potatoes, Main Courses, Pizza, Salads, Sandwiches, Soup and Toppings & Ingredients.

### Pan American Health Organisation nutrient profile model

There are few recommended dietary guidelines for restaurant menu items. Those that exist typically focus on whole meals rather than individual menu items^(29)^. Instead, we used the Pan American Health Organisation (PAHO) Nutrient Profile Model (NPM) to calculate which menu items contained excess levels of Na, total fat, saturated fat or free sugars (Table 1)^(30)^. These criteria are largely similar to the WHO’s healthy diet guidelines^(31)^. We applied these criteria to items from menus in both countries. As information on free sugar content was not available in our data, we made assumptions about the relationship between free sugars and total sugars (see online Supplemental Appendix Table S1). The assumptions were also based on the PAHO NPM, with modifications to accommodate restaurant menu items, as ingredient information is typically not provided by restaurants^(30)^.

**Table 1:** PAHO NPM criteria: items high in Na, total fat, saturated fat and free sugars

Nutrient	Na	Total fat	Saturated fat	Free sugars
Criteria	≥1 mg Na/kcal	≥30 % of energy from total fat	≥10 % of energy from total fat	≥10 % of energy from free sugars

### Statistical analysis

We used linear mixed models with random intercepts for adult and children’s menu items separately, to predict differences between countries in energy (kcal), fat (g), saturated fat (g), carbohydrates (g), sugars (g), protein (g) and Na (mg) per item served by chain restaurants. Random intercepts were used to account for restaurant- and country-level clustering. We controlled for restaurant type, food category, limited time offer, regionally offered items and shareable status. We also calculated the proportions of adult and children’s menu items that exceeded recommended levels of Na (mg), total fat (g), saturated fat (g) or free sugars (g) in both countries based on the PAHO NPM criteria.

In sensitivity analyses, we first tested if the results were robust to differences in what restaurants were present in the UK and the USA. We restricted our analyses to chain restaurants operating in both countries to provide a like-for-like rather than full landscape comparison (see online Supplemental Appendix Fig. S2). Second, we calculated the odds ratio of a USA item being high in Na, total fat, saturated fat or free sugars compared with a UK item using mixed effect logistic regressions, adjusted for restaurant – and item-level covariates (see online Supplemental Appendix Table S3). This was to tease out the potential effect of these covariates on proportions of items high in Na, total fat, saturated fat or free sugars.

All statistical analyses were performed in R (version 4.0.2; R Foundation for Statistical Computing).

## Results

Out of the 100 largest chains, forty-two chains in the UK and ninety-six chains in the USA provided some form of energy and nutrition information online in 2018. Energy and nutritional information of menu items served by these chains were included. Across these chains, 10 782 menu items were served in the UK and 30 120 in the USA. Compared with the UK, adult menu items in the USA were more likely to be served by fast-food restaurants, described as shareable, or were regionally offered (Table 2). Similarly, children’s menu items in the USA were more likely to be served by fast-food restaurants and described as limited time or regionally offered. The distribution of items across food categories also varied. For example, 30·5 % and 40·5 % of USA adult and children’s menu items were beverages, while only 21·9 % and 3·9 % were beverages in the UK. The availability of energy and nutrient information of these menu items has been described elsewhere^(25,32)^.

**Table 2:** Restaurant- and item-level characteristics

.	Adult menu items					Children’s menu items				
	UK *n* 9852		USA *n* 28 229		*P*-value*	UK *n* 930		USA *n* 1891		*P*-value*
	*n*	%	*n*	%		*n*	%	*n*	%	
Restaurant type										
Fast-casual	2878	29·2	4972	17·6		10	1·1	533	28·2	
Fast-food	2552	25·9	14 345	50·8	<0·001	105	11·3	533	28·2	<0·001
Full-service	4422	44·9	8912	31·6		815	87·6	825	43·6	
Shareable										
Non-shareable	9603	97·5	27 351	96·9	0·004	930	100	1884	99·6	0·146
Shareable	249	2·5	878	3·1		0	0	7	0·4	
Food category										
Appetizers and sides	1141	11·6	1910	6·8		334	35·9	176	9·3	
Baked goods	539	5·5	938	3·3		35	3·8	2	0·1	
Beverages	2155	21·9	8609	30·5		36	3·9	765	40·5	
Burgers	364	3·7	701	2·5		37	4	65	3·4	
Desserts	677	6·9	1340	4·7	<0·001	92	9·9	87	4·6	<0·001
Main courses	1206	12·2	3543	12·6		173	18·6	367	19·4	
Fried potatoes	206	2·1	316	1·1		77	8·3	46	2·4	
Pizza	1514	15·4	1610	5·7		28	3	25	1·3	
Salads	284	2·9	868	3·1		26	2·8	34	1·8	
Sandwiches	668	6·8	3326	11·8		30	3·2	114	6	
Soup	192	1·9	570	2		3	0·3	19	1	
Toppings and Ingredients	906	9·2	4498	15·9		59	6·3	191	10·1	
Regionally offered										
Non regionally offered item	9801	99·5	27 161	96·2	<0·001	929	99·9	1863	98·5	0·001
Regionally offered item	51	0·5	1068	3·8		1	0·1	28	1·5	
Limited time offer										
Non limited time offer	9642	97·9	27 593	97·7	0·506	930	100	1877	99·3	0·019
Limited time offer	210	2·1	636	2·3		0	0	14	0·7	

* *P*-value derived from *χ*^2^ tests.

### Differences in mean absolute energy and nutrient values of adult menu items

**Fig. 1:**
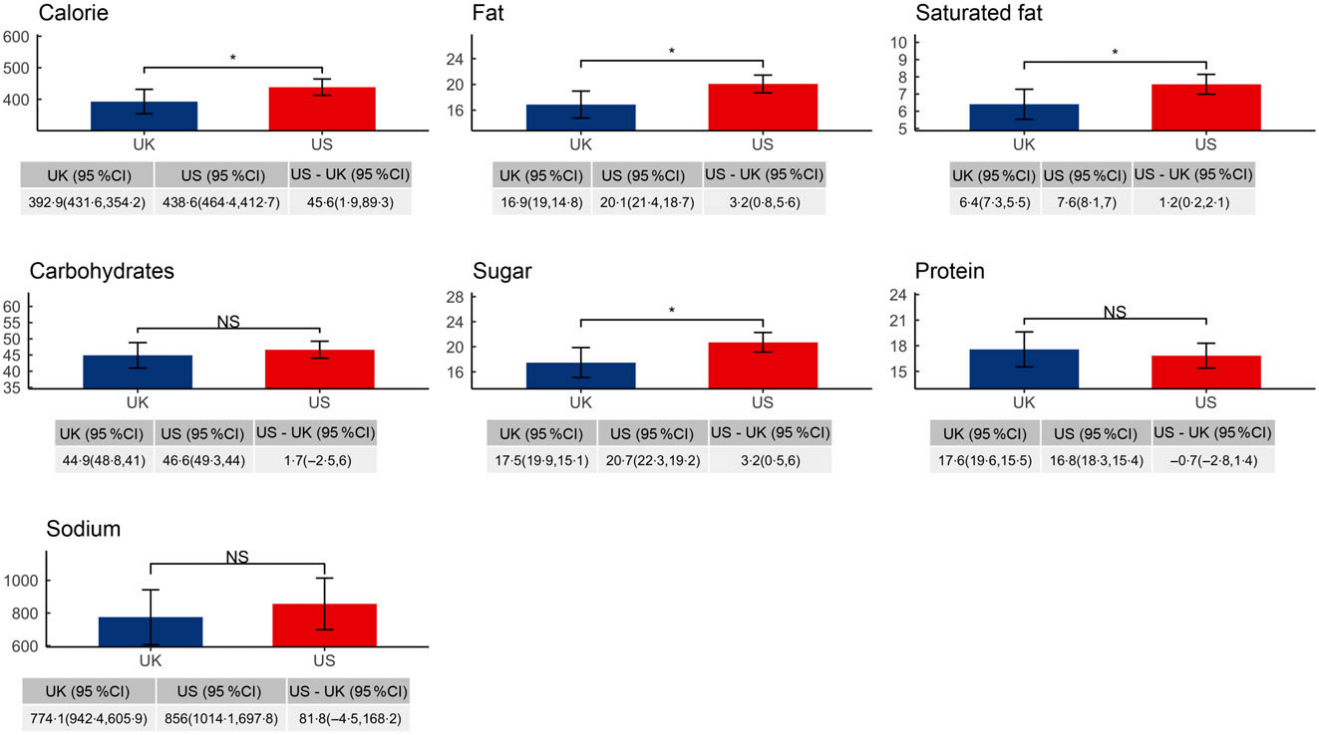
Predicted energy and nutrient values per item in adult menu items, by country. Adjusted for restaurant type, food group, limited time offer, regionally offered status and shareable status. *P < 0·05. NS, not statistically significant

Figure 1 shows the predicted mean absolute energy and nutrient values per item for adult menu items in each country after adjusting for restaurant and menu-level covariates. Mean absolute predicted energy and nutrient values were higher in USA than UK for energy, fat, saturated fat and sugars. The mean absolute per-item differences between the USA and the UK were 45·6 kcal for energy (95 % CI (1·9, 89·3)), 3·2 g for fat (95 % CI (0·8, 5·6)), 1·2 g for saturated fat (95 % CI (0·2, 2·1)) and 3·2 g for sugars (95 % CI (0·5, 6·0)). Differences in mean absolute per-item differences between the USA and UK were not statistically significant for carbohydrates, protein and Na. Results from unadjusted analyses are shown in the Supplemental Appendix Table S4.

### Differences in mean absolute energy and nutrient values of children’s menu items

**Fig. 2:**
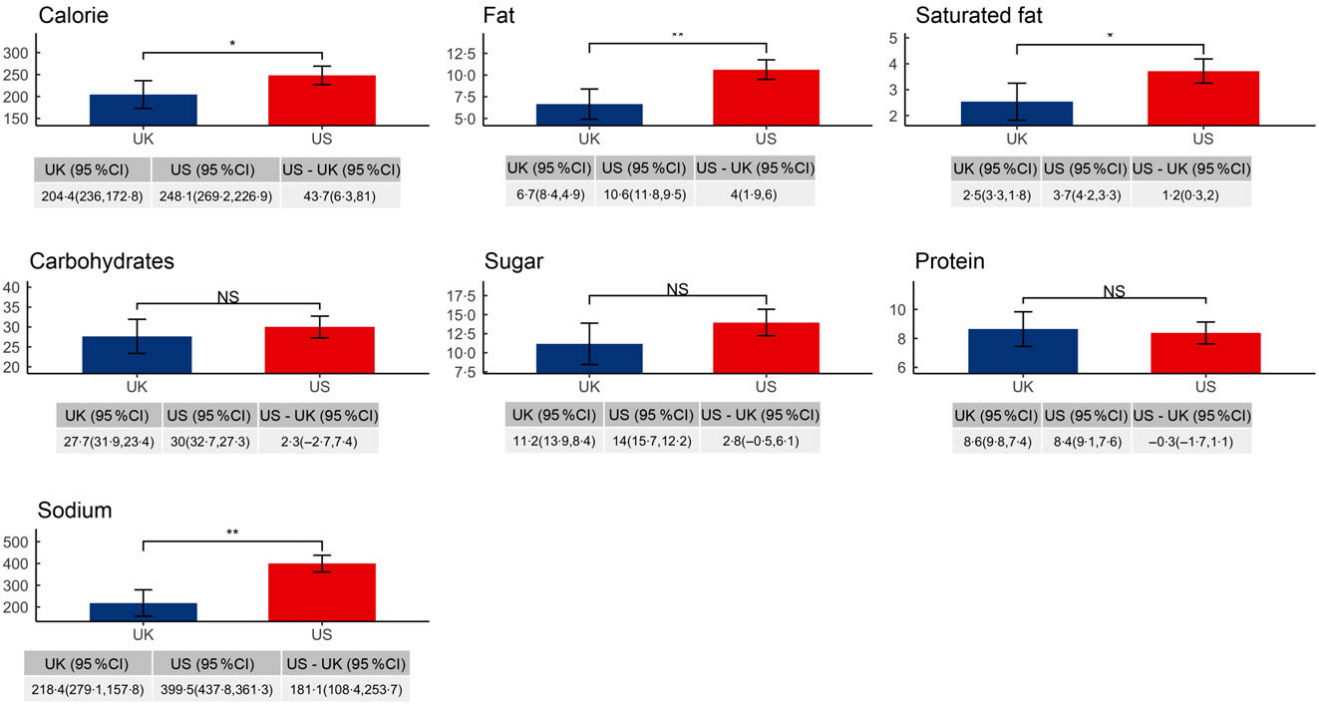
Predicted energy and nutrient values per item in children’s menu items, by country. Adjusted for restaurant type, food group, limited time offer, regionally offered status and shareable status. **P < 0·001, *P < 0·05. NS, not statistically significant

As shown in Fig. 2, among children’s menu items, after adjustment for restaurant and menu-level covariates, mean absolute predicted energy, fat, saturated fat and Na values were higher in USA than UK. Compared with an average children’s menu item from a large chain restaurant in the UK, an average children’s menu item from a large chain restaurant in the USA contained more energy by 43·7 kcal (95 % CI (6·3, 81)), more fat by 4·0 g (95 % CI (1·9, 6·0)), more saturated fat by 1·2 g (95 % CI (0·3, 2·0)) and more Na by 181·1 mg (95 % CI (108·4, 253·7)). Results from unadjusted analyses are shown in the Supplemental Appendix Table S4.

In the sensitivity analysis, we analysed nutritional differences of menu items served by chains operating in both countries and found that the results were largely similar. Across these chains, energy and nutrient values were higher in the USA, except for protein and saturated fat (Supplementary Appendix Figure S2).

### Proportions of menu items in excess of recommended nutrient levels

**Fig. 3:**
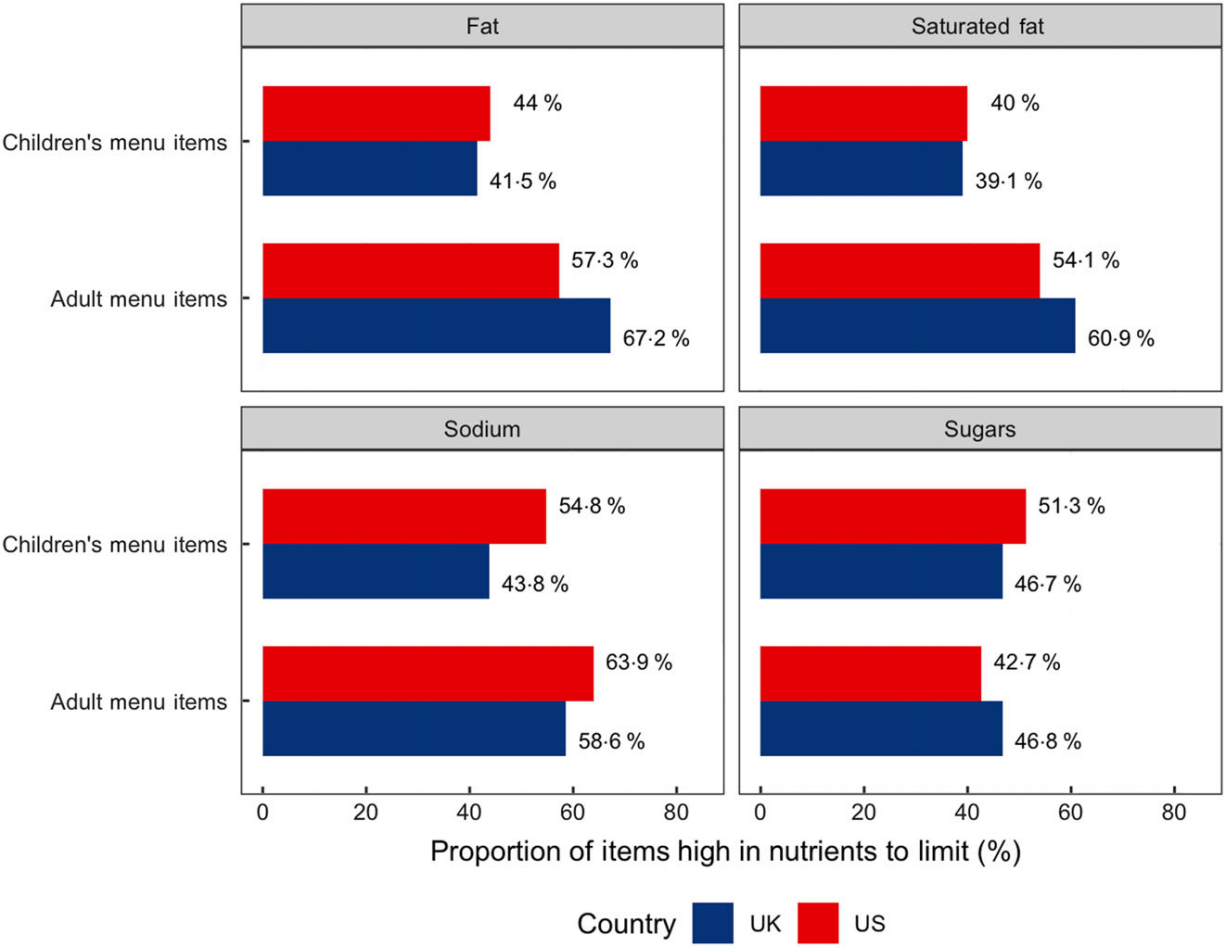
Proportion of children’s and adult’s menu items high in fat, saturated fat, sodium and sugars, by country

As shown in Fig. 3, in the USA, higher proportions of children’s menu items contained excess levels of fat, saturated fat, Na or sugars according to the PAHO recommendations compared with that in the UK. In contrast, adult menu items from the USA had lower proportions of items that exceeded nutritional recommendations, except for Na. Overall, 96·8 % of UK and 95·8 % of USA menu items exceeded recommended levels for at least one of Na, fat, saturated fat or sugars according to the PAHO NPM. In the sensitivity analysis, we estimated the odds of a menu item being high in nutrients to limit in the two countries, adjusted for restaurant- and item-level characteristics (see online Supplemental Appendix Table S3), and results were consistent.

## Discussion

### Summary of findings

Our study was the first to describe and compare the energy and nutritional composition of adults and children’s menu items served by large chain restaurants in the UK and the USA. We sourced information on more than 40 000 menu items served by 138 large restaurant chains. After adjustment for restaurant- and item-level characteristics, adult menu items in the USA had higher absolute energy, fat, saturated fat and sugar levels on average than those in the UK. USA children’s items had higher absolute energy, fat, saturated fat and Na than those in the UK. USA children’s items contained 80 % more Na and about 50 % more saturated fat than the UK ones. We used a recognised international nutrient profiling model to assess nutrient content (relative to energy) and identify items high in nutrients to limit. More than 95 % of items were high in at least one nutrient to limit and 39–67 % of adult and children’s items from each country were high in each nutrient. Higher proportions of children’s menu items contained excess levels (relative to energy) of Na, fat, saturated fat and free sugars in the USA, compared with the UK. The reverse was seen for adult items: higher proportions of adult menu items in the UK had excess (relative to energy) levels of fat, saturated fat and free sugars, compared with those in the USA.

### Strength and limitations

Our study is the first to assess energy and nutritional differences across whole menus of all types of large chain restaurants in the UK and the USA. To the best of our knowledge, it is also the largest to date to investigate the nutritional differences between restaurant menu items across countries.

In terms of limitations, we only examined menu items served by restaurants in the top 100 for sales in each country that published nutritional information online in 2018. Meals from smaller chains and independent restaurants may also contain high levels of energy and other nutrients to limit; however, they were not included in our study^(33,34)^. Smaller chains and independent restaurants typically do not provide nutritional information online. This (and the proportion of large restaurants providing information on the websites in the UK *v*. USA) may reflect differences in menu labelling policy – in the USA chains with twenty or more outlets have had to provide menu labelling since 2014; in the UK chains with 250 or more employees have had to do so since April 2022^(35,36)^. However, the results from the sensitivity analysis where we compared restaurants that operate in both countries indicate the nutritional differences were robust regardless of whether we included the same or all restaurants from each country.

Furthermore, the primary outcomes (e.g. the mean energy/nutrient per item) were not weighted by sales, due to the lack of restaurant sales data. Therefore, the averages of energy and nutritional values focused on what was available on the menu, rather than what was purchased and/or consumed. As portion sizes were largely missing (∼67 %), we also could not investigate between-country differences in portion sizes.

Moreover, we standardised pizza portions based on the approach used by one chain, which may not accurately reflect actual pizza portions. However, as the same set of standardisation rules were applied to both USA and UK pizza items, we anticipated the results to be robust against different definitions of a pizza portion.

Lastly, the PAHO NPM includes criteria for identifying items high in free sugars, regarding which we had no data. The assumptions we used to convert reported total sugars into estimated free sugars were based on the PAHO NPM. However, their method for estimating free sugars from total reported sugar requires a list of ingredients for each product and such data are not available for restaurant foods. Our modified method did not take into account the sophisticated nature of the relationship between free sugars and total sugars, and there could be an overestimation of proportions of items that contained excess levels of free sugars. While there were many dietary guidelines to choose from, the PAHO guideline is designed for individual food and drink products (rather than whole diets) and allowed menu item-level comparison. It is broadly consistent with the WHO international dietary guideline provided for whole diets^(31)^.

### Interpretation of results and implications for policy

#### Proportion of menu items with excessive levels of Na, total fat, saturated fat or free sugars

In both the UK and USA, government interventions designed to improve public health through affecting change in the food system have been and are being introduced. These include voluntary targets (in both countries), such as salt or Na content reduction, and mandatory regulations, such as menu calorie labelling (currently mandatory in the USA, and implemented in the UK in April 2022) and advertising restrictions on less healthy foods and menu items on television (proposed in the UK)^(37–41)^. Despite these efforts, our study found over 95 % of menu items to have excess levels of Na, total fat, saturated fat or free sugars in both the USA and the UK, as of 2018. This is broadly consistent with previous findings using different dietary recommendations^(8,11)^. These findings suggest that the current policies designed to improve the healthfulness of restaurant foods may not yet be achieving their intended effects. Policymakers should consider additional ways to ensure it is easy to choose healthy out-of-home options. For example, the UK’s National Food Strategy recommends introducing a sugar and salt reformulation tax in restaurants, to incentivise recipe reformulation^(42)^. Other fiscal policies, such as the UK’s Soft Drinks Industry Levy, have shown high effectiveness in promoting reformulation^(43)^.

#### Variations in energy and nutrient content of restaurant menu items

In this study we found considerable variation in the energy and nutrient content of restaurant menu items, which is in line with previous multi-country studies^(19,44,45)^. Large chain restaurants in the UK tended to offer food and beverages that were lower in absolute energy, fat and saturated fat compared with those in the USA. UK adult menu items were also lower in sugars compared with USA ones and children’s items lower in Na. This might partly be explained by differing portion sizes between the two countries. However, if different portion sizes were the full explanation, differences in other nutrients would be proportional to those in energy and they were not. Differences in food composition or preparation may also play a role in the between-country differences we found. In terms of macro-level differences in composition, it may be, for example, that main courses in the USA typically include a side, whilst in the UK they do not. At a more micro-level, chain restaurants appear to use different ingredients, even for the same menu item in different countries, with, for example, the USA ingredient list for a McDonald’s Big Mac being considerably longer than the UK equivalent (see online Supplemental Appendix Table S5). There may also be systematic differences in reliance on processed and ultra-processed foods.

Our results set a benchmark for future monitoring of restaurant menu items and highlight room for improvement for large chain restaurants in the USA in particular. Subject to consumer acceptability, the energy, fat and saturated fat content of menu items in the USA could potentially match the equivalent UK energy and nutrient levels. Future studies exploring category-specific differences could also shed light on which food categories to prioritise.

#### Difference in energy and nutrient content of children’s menu items

Between-country differences in energy and nutrients were evident among children’s menu items, with an average children’s menu item in the USA having an additional 43·7 kcal and 181·1 mg of Na in the USA than in the UK. As the frequency of eating outside the home is also associated with less healthy dietary intake in children (as well as in adults), children’s meals are a target for reducing childhood obesity^(46,47)^. Previous modelling studies predicted that a relatively small reduction in daily energy intake could be sufficient to reverse the trend of increasing body weight among children^(48,49)^. As such, small, gradual and consistent improvements in children’s menu items may help tackle childhood obesity. This seems particularly achievable in the USA if item composition moves further towards that seen in the UK.

#### Restaurant menu items exceeding Pan American Health Organisation Nutrient Profile Model nutrient levels

Despite the lower absolute values of energy and nutrients studied in UK items, our results indicate that UK adult menu items were more likely to exceed recommended fat, saturated fat and sugar levels, based on PAHO NPM criteria than those in the USA. At face value, these results seem to contradict one another. However, the PAHO NPM criteria examine nutrient content relative to energy content. It seems that a greater proportion of energy in adult menu items come from fat, saturated fat and sugars in the UK than in the USA. In contrast, non-sugar carbohydrates might make up a higher proportion of energy in the USA. Although a few national initiatives (e.g. sugar reduction program in 2016) have been introduced in the UK to incentivise reformulation in the chain restaurant sector, the effect of these programmes may have not been fully realised at the time of our data collection^(50)^. Continued monitoring of restaurant foods would help understand the potential effect of existing and any new government programmes, and build the evidence for how these types of population-level interventions work in practice^(27,51)^.

## Conclusions

In this study, we found that menu items served by large chain restaurants had higher absolute levels of energy, fat and saturated fat in the USA than in the UK. USA adult menu items also had higher sugar content compared with the UK. Between-country differences were prominent in children’s menu items, especially for Na and saturated fat. However, as more than 95 % of all items in both countries were considered to have high levels of at least one nutrient to limit in the PAHO NPM, improvements in the nutritional composition of restaurant menu items are needed in both countries.

## Supplementary material

For supplementary material accompanying this paper visit https://doi.org/10.1017/S1368980022001379. click here to view supplementary material
