# Supplementary material for: Differences in energy and nutrient content of menu items served by large chain restaurants in the USA and the UK in 2018
Source: Public Health Nutr. 2022 Jun 1;25(10):2671–9. doi: 10.1017/S1368980022001379 (PMC9991847; doi:10.1017/S1368980022001379)
Supplement: Supplementary file 1 [file S1368980022001379sup001.docx]

SUPPLEMENTARY APPENDIX

Differences in Energy and Nutrient Content of Menu Items Served by Large Chain Restaurants in the US and the UK

**Table S1** **Assumptions on the relationship between sugar and free sugar**

If the menu item contained 0g of sugar or is fresh fruit or unflavoured milk (identified through keywords listed below), then the free sugar content was 0g. If a menu item name contained “milk” or “fruit”, but was not fresh fruit or unflavoured milk, we calculated the free sugars as 75% of declared total sugars. For all others, we assumed all sugars were free sugars.

| If the menu item… | Free sugar | Keywords |
| --- | --- | --- |
| Contains 0g of sugar | 0g |  |
| Is fresh fruit or unflavoured milk | 0g | Fresh fruit, fruit salad, skimmed milk, whole milk, semi-skimmed milk |
| Contains ‘milk’ or ‘fruit’ | 75% of declared total sugars | milk, fruit |
| All others | Total sugars |  |

**Figure S2 Restaurants Available in Both Countries, All Menu Items**

**Restaurants available in both countries:** Burger King, Domino’s, KFC, Krispy Kreme, McDonald’s, Papa John’s, Panda Express, Pizza Hut, Starbucks, Subway


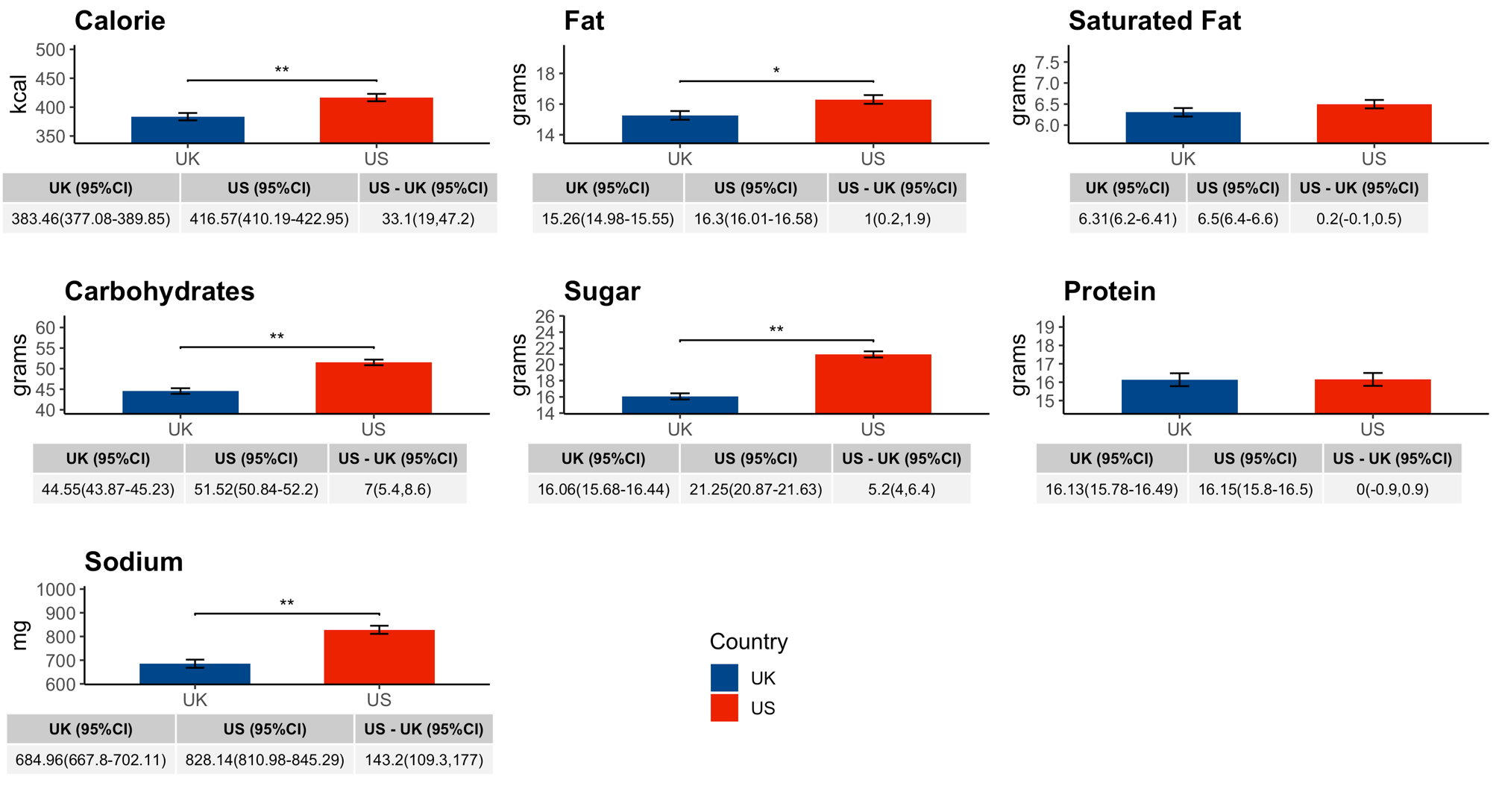


**Figure S2** Predicted energy and nutrient values in all menu items among restaurants that operate in both countries. Adjusted for restaurant type, food group, limited time offer, regionally offered items, and shareable status.

**Table S3 Odds ratios of a menu item high in sodium, fat, saturated fat, or sugar***

|  | **Adult menu items** | | **Children’s menu items** | |
| --- | --- | --- | --- | --- |
| Odds ratio of a US menu item high in the nutrient compared a UK menu item | Unadjusted odds | Adjusted odds ratio* | Unadjusted odds ratio | Adjusted odds ratio* |
| Sodium | 1.3 (1.2, 1.3) | 2.9 (2.2, 3.8) | 1.6 (1.3, 1.8) | 2.8 (1.9, 4.1) |
| Fat | 0.5 (0.5, 0.6) | 0.8 (0.6, 1.1) | 1.1 (0.9, 1.3) | 1.7 (0.9, 3.5) |
| Saturated fat | 0.4 (0.4, 0.4) | 0.6 (0.4, 0.9) | 0.9 (0.8, 1.0) | 1.6 (0.8, 3.2) |
| Sugar | 0.6 (0.5, 0.6) | 0.6 (0.5, 0.8) | 2.3 (1.9, 2.7) | 1.3 (0.8, 2.1) |

* Mixed logistic models with random intercepts, adjusted for restaurant type, food group, limited time offer, regionally offered items, and shareable status.

**Table S4 Crude Mean Energy and Nutrient Values (SD) of Adult and Children’s Menu Items**

| Country | Adult/Children | Energy(kcal) | Fat (g) | Saturated Fat (g) | Carbohydrates(g) | Sugar(g) | Protein (g) | Sodium(g) |
| --- | --- | --- | --- | --- | --- | --- | --- | --- |
| UK | Adult | 441.12(4.03) | 20.03(0.22) | 7.37(0.08) | 45.07(0.40) | 14.33(0.17) | 18.94(0.27) | 710.51(8.44) |
| UK | Children | 208.61(5.52) | 7.50(0.28) | 2.69(0.11) | 25.48(0.65) | 7.11(0.33) | 8.14(0.31) | 233.13(9.34) |
| US | Adult | 421.10(2.93) | 19.09(0.17) | 7.13(0.07) | 45.89(0.33) | 22.09(0.23) | 16.30(0.16) | 790.13(7.12) |
| US | Children | 229.84(5.88) | 9.42(0.37) | 3.41(0.13) | 30.15(0.70) | 15.85(0.51) | 7.69(0.25) | 377.50(16.01) |

**Table S5 Ingredient lists for the Big Mac Sauce (UK & US)**

| UK ^a^ | US ^b^ |
| --- | --- |
| 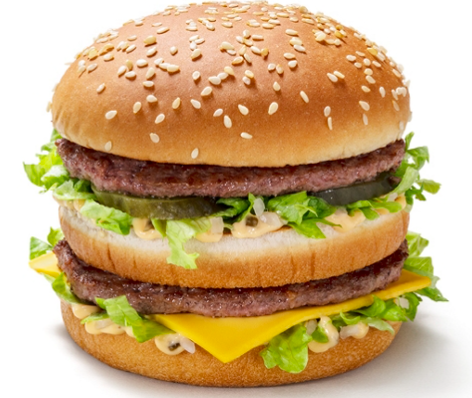 | 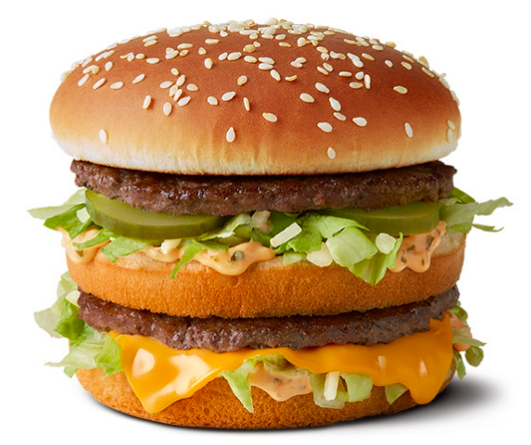 |
| 508 kcal | **550 Cal.** |
| Big Mac Sauce  Water, Rapeseed Oil, Gherkin, Spirit Vinegar, Sugar, Modified Maize Starch, Allergen Ingredient: Free Range EGG Yolk, Spices (contain Allergen Ingredient: MUSTARD), Salt, Glucose-Fructose Syrup, Thickener (Xanthan Gum), Natural Flavourings, Firming Agent (Calcium Chloride). | Big Mac Sauce  Soybean Oil, Sweet Relish (Diced Pickles, Sugar, High Fructose Corn Syrup, Distilled Vinegar, Salt, Corn Syrup, Xanthan Gum, Calcium Chloride, Spice Extractives), Water, Egg Yolks, Distilled Vinegar, Spices, Onion Powder, Salt, Propylene Glycol Alginate, Garlic Powder, Vegetable Protein (Hydrolyzed Corn, Soy and Wheat), Sugar, Caramel Color, Turmeric, Extractives of Paprika, Soy Lecithin. |

^a^ source: <https://www.mcdonalds.com/gb/en-gb/product/big-mac.html>, accessed on Sep 30, 2021

^b^ source: <https://www.mcdonalds.com/us/en-us/product/big-mac.html>, accessed on Sep 30, 2021
